# Ferroptosis in sepsis induced acute lung injury/acute respiratory distress syndrome (ALI/ARDS): a potential therapeutic strategy

**DOI:** 10.3389/fimmu.2025.1689155

**Published:** 2025-11-26

**Authors:** Linlin Wang, Wenzhe Zhang, Xiujing Feng

**Affiliations:** 1 Department of Critical Care Medicine, The First Affiliated Hospital of Shandong First Medical University & Shandong Provincial Qianfoshan Hospital, Shandong Institute of Anesthesia and Respiratory Critical Medicine, Jinan, Shandong, China; 2 School of Clinical and Basic Medical Sciences, Shandong First Medical University& Shandong Academy of Medical Sciences, Jinan, Shandong, China; 3 Key Laboratory of Endocrine Glucose & Lipids Metabolism and Brain Aging, Ministry of Education; Department of Endocrinology, Shandong Provincial Hospital Affiliated to Shandong First Medical University, Jinan, Shandong, China

**Keywords:** ferroptosis, sepsis induced acute lung injury/acute respiratory distress syndrome, therapeutic strategy, immune regulation, oxidative stress

## Abstract

Sepsis induced acute lung injury/acute respiratory distress syndrome (ALI/ARDS) remains a devastating complication of sepsis, marked by uncontrolled pulmonary inflammation, alveolar–capillary barrier disruption, and high mortality. Despite advances in supportive care, no targeted medicines are currently available. Ferroptosis is an iron-dependent and non-apoptotic form of cell death characterized by the iron induced accumulation of lipid reactive oxygen species (ROS). Emerging evidence indicates that ferroptosis is involved in the progression of sepsis induced ALI/ARDS, although the mechanism of action of ferroptosis in sepsis induced ALI/ARDS is still poorly understood. This mini-review summarizes the mechanism of ferroptosis action on sepsis induced ALI/ARDS, with particular focus on immune dysregulation, endothelial/epithelial dysfunction, and oxidative stress. We highlight key molecular pathways, including glutathione peroxidase 4 (GPX4) inactivation, iron metabolism disruption, and lipid peroxidation cascades, supported by both preclinical studies and emerging clinical correlates. Furthermore, discuss the potential therapeutic approaches currently used to treat ARDS. This review also discusses major challenges to clinical translation and highlights further directions for the treatment and prevention of sepsis induced ALI/ARDS.

## 1 Introduction

Sepsis, a prevalent critical condition in clinics, continues to be the leading cause of death from infections and a global healthcare issue. Among the organs susceptible to the harmful effects of sepsis, the lungs are notably the most frequently affected. Consequently, patients with sepsis are predisposed to developing acute lung injury (ALI), and in severe cases, acute respiratory distress syndrome (ARDS) with a high mortality rate, exceeding 30% (1, 2). Despite significant advance in the understanding and management of ALI/ARDS, there remains a substantial lack of drugs capable of effectively treating ALI/ARDS induced by sepsis due to limited research on its underling mechanism.

Ferroptosis was proposed by Dixon (3) in 2012, driven by iron-dependent phospholipid peroxidation, is regulated by multiple cellular metabolic pathways, including redox homeostasis, iron handling, and metabolism of amino acids, lipids and sugars, in addition to various signaling pathways relevant to disease. Numerous organ injuries and degenerative pathologies are driven by ferroptosis, such as neurodegenerative diseases (4), endocrine diseases and cancer (5, 6). In recent years, the role of ferroptosis in sepsis induced ALI/ARDS has received more research attention (7, 8). The research on the role of ferroptosis in sepsis induced ALI/ARDS has progressed rapidly. Studies has highlighted the importance of immune dysfunction, oxidative stress, and barrier disruption in its pathogenesis. This mini-review integrates the latest (2020–2025) advances in understanding ferroptosis in sepsis-induced ALI/ARDS, emphasizing the role of ferroptosis and the potential therapeutic strategies by targeting ferroptosis. Moreover, we discuss its significance in the treatment of sepsis-induced ALI/ARDS and provide new directions for the treatment and prevention of sepsis-induced ALI/ARDS by targeting ferroptosis.

## 2 Ferroptosis in sepsis-induced ALI/ARDS

### 2.1 Molecular mechanism of ferroptosis

**Figure 1:**
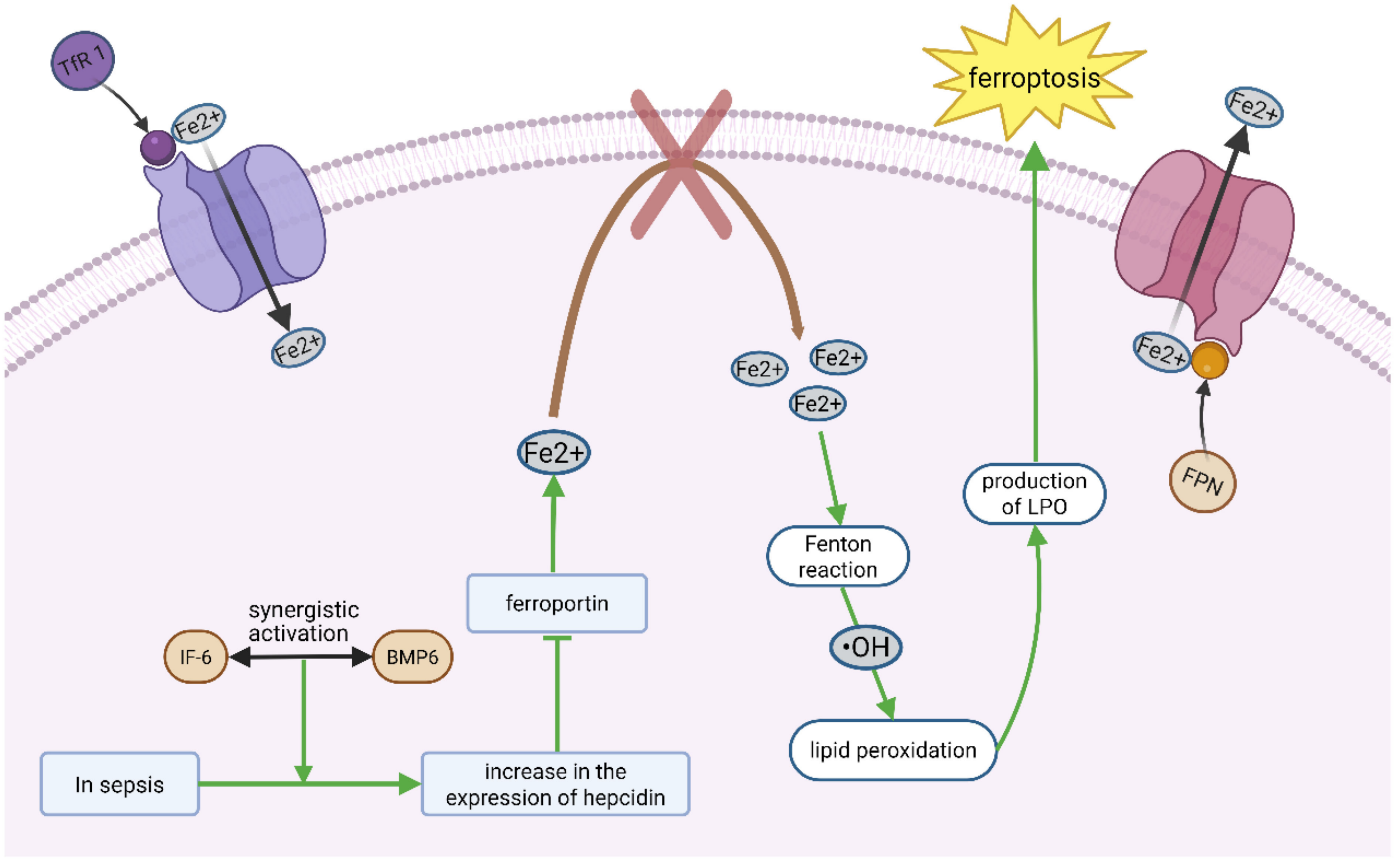
Ferroptosis mainly involves the disruption of redox homeostasis. Transferrin receptor 1 (TfR 1) mediates iron entry, whereas iron transporter (FPN) mediates iron output. Under sepsis conditions, the synergistic activation of IL-6 (inflammatory pathway) and Bmp6 (iron-sensitive pathway) triggers an increased expression of hepcidin, which inhibits the function of the iron export protein ferroportin. As a result, iron is not properly released from the lung cells, causing iron accumulation within the cells. The accumulated free iron generates highly reactive radicals through the Fenton reaction, which initiate and catalyze chain reactions of lipid peroxidation, producing a large amount of lipid peroxides (LPO), which then directly damages the cellular lipid membrane and becomes a significant factor triggering iron-induced cell death.

Intracellular labile iron (Fe²^+^) is central to ferroptosis, catalyzing the Fenton reaction to generate hydroxyl radicals (•OH), which initiate lipid peroxidation (9). Thus, ferroptosis fundamentally reflects a breakdown of redox homeostasis. Iron balance is tightly regulated by uptake, export, and storage. Transferrin receptor 1 (TfR1) mediates iron entry, and its upregulation in sepsis-induced ARDS correlates with ferroptosis markers (10). Conversely, ferroportin (FPN), the only known iron exporter, is downregulated in sepsis due to hepcidin overexpression, trapping iron intracellularly (11). In septic lung injury, ferroptosis amplifies tissue damage through iron-dependent lipid peroxidation in alveolar epithelial cells. Recent studies reveal that sepsis-induced hypoxia upregulates hypoxia-inducible factor-1α (HIF-1α), which increases cellular iron uptake via transferrin receptor 1 (TfR1). Of course, hepcidin expression requires synergistic activation by IL-6 (inflammatory pathway) and BMP6 (iron-sensing pathway), which exhibits elevated in sepsis-induced ARDS patients (12). Moreover, Ferritin, which sequesters iron, undergoes degradation via ferritinophagy (a selective autophagy process) in sepsis-induced ARDS. Ferritinophagy mediated by Nuclear receptor coactivator 4 (NCOA4), which first precisely recognizes and binds to ferritin and recruits and directs the autophagy machinery to enclose ferritin into autophagosomes, ultimately transporting it to lysosomes for degradation, thereby releasing the free iron for cellular use (13). In addition, ferroptosis activators such as erastin and RSL3 increase intracellular iron accumulation by impairing antioxidant systems (14) (**Figure 1**). The glutathione (GSH)-GPX4 axis is the primary defense against ferroptosis. GPX4 reduces LPO to non-toxic alcohols by using GSH, but this system is compromised in sepsis-induced ARDS. System Xc^-^ (a cystine/glutamate antiporter) supplies cystine for GSH synthesis. In sepsis, TNF-α and IL-1β downregulate its subunit SLC7A11, reducing GSH levels by 40%–60% in lung tissues (15, 16). Concurrently, sepsis depletes GSH by downregulating the xCT transporter, impairing GPX4 activity (61). This dual hit iron overload + antioxidant failure explains the prominence of ferroptosis in septic lungs. While these mechanisms are increasingly well-characterized, key questions remain regarding cell-type specific responses and the temporal sequence of these interactions during sepsis progression. The above results suggest that ferroptosis is involved in the pathogenesis of lung injury, which will provide a new theoretical basis for the clinical treatment of ARDS. However, no clinical studies have examined the association of these ferroptosis indicators with severity and prognosis of ARDS.

### 2.2 Endothelial/epithelial cell damage

**Figure 2:**
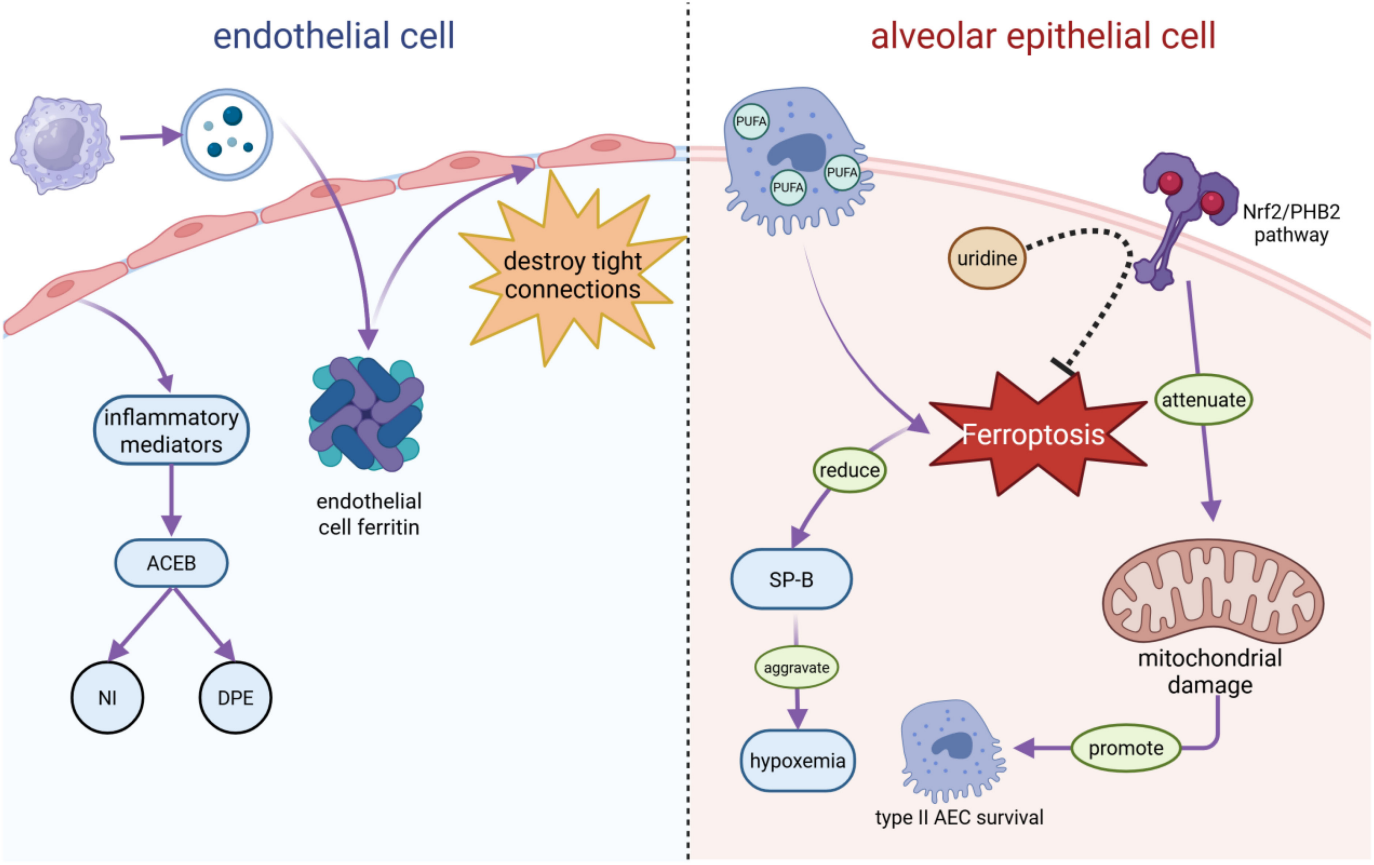
S-ALI causes endothelial and epithelial cell damage. In sepsis-induced ARDS, endothelial cells secrete inflammatory mediators that disrupt the alveolar-capillary endothelial barrier (ACEB), triggering neutrophil infiltration (NI) and diffuse pulmonary edema (DPE). Meanwhile, macrophage-derived extracellular vesicles induce an increase in endothelial cell ferritin, which, in turn, destroys tight connections and increases the alveolar capillary permeability. In alveolar epithelial cells (AECs), type II AECs are prone to iron apoptosis owing to their abundance of polyunsaturated fatty acids (PUFAs), which reduce surfactant protein B (SP-B) and aggravate hypoxemia. The activation of its Nrf2/PHB2 pathway attenuates mitochondrial damage, promotes type II AEC survival, and prevents progression to ALI in sepsis mice. In addition, uridine can inhibit iron apoptosis in macrophages by activating the Nrf2-signaling pathway.

Sepsis-induced ARDS releases abundant inflammatory mediators that disrupt the structural integrity of the alveolar–capillary endothelial barrier, causing neutrophil infiltration (NI) and diffuse pulmonary edema (DPE) (17, 18). Macrophage-derived extracellular vesicles promote endothelial ferroptosis, further compromising barrier integrity (19). Through LPO-mediated membrane injury, ferroptosis damages tight junctions, increasing alveolar-capillary permeability. Notably, single-cell RNA sequencing identified endothelial ferroptosis as an early sepsis-induced ARDS event, preceding edema formation (20). Extracellular vesicles also play protective roles: for example, ADSC-derived exosomal miR-125b-5p mitigates inflammation-induced pulmonary microvascular endothelial ferroptosis in sepsis-induced ARDS by regulating Keap1/Nrf2/GPX4 expression (21). Type II alveolar epithelial cells (AECs), rich in polyunsaturated fatty acids (PUFAs), are particularly vulnerable to ferroptosis. Their loss impairs surfactant protein B production, worsening hypoxemia (22). Activating the Nrf2/PHB2 pathway in type II AECs reduces mitochondrial injury caused by sepsis in mice, thereby promoting the survival of type II AECs and preventing progression to ALI (23). Additionally, neutrophil extracellular traps (NETs) exacerbate sepsis-induced ARDS by inducing ferroptosis in alveolar epithelial cells (24). (**Figure 2**). Above data suggests that ferroptosis induced endothelial and epithelial cell ferroptosis damage play an important role in sepsis induced ALI/ARDS, protecting against its ferroptosis will supply potential therapeutic strategy for this disease.

### 2.3 Immune regulation

Ferroptosis is closely intertwined with immune regulation. Ferritin degradation promotes the release of damage-associated molecular patterns, including HMGB1 and ATP, which in turn activate multifunctional nanozyme systems such as MET-CMS@FeTA (MCMSFT) and the NLRP3 inflammasome, while also driving macrophage polarization (25). From 2021–2023, studies have revealed that ferroptosis-derived LPO directly activates NLRP3, triggering IL-1β and IL-18 secretion, which further enhances ferroptosis through SLC7A11 downregulation (7, 26, 27). Also, pro-inflammatory signals (TNF-α/IL-1β) activate core signaling pathways (mainly NF-κB/MAPK), jointly increasing cellular sensitivity to ferroptosis at multiple levels. The main targets include: downregulation of GSH synthesis genes, upregulation of ACSL4/LPCAT3, and regulation of iron storage and uptake (28).

In septic mice, dysregulated inflammation can cause widespread damage to alveolar epithelial and microvascular endothelial cells, resulting in pulmonary edema and excessive neutrophil infiltration. Sepsis disrupt immune homeostasis, with pulmonary macrophages differentiating into distinct subtypes to regulate inflammation at different stages. Moreover, previous research has shown that mediating the Nrf2 pathway can inhibits ferroptosis in macrophages, thereby exerting a protective effect against sepsis-induced ARDS (24). Moreover, Ferrostatin-1, the inhibitor of ferroptosis, was found to rescue the downregulation of ferroptosis markers including cysteine/glutamate transporter (SLC7A11) and GPX4 in sepsis induced ALI/ARDS (29). Also, STING promotes sepsis-induced ALI/ARDS by inducing macrophage ferroptosis in a cGAS- and interferon-independent manner. Mechanistically, Q237, E316, and S322 in the CBD domain of STING are critical binding sites for the interaction with the coiled-coil domain of NCOA4. Their interaction not only triggers ferritinophagy-mediated ferroptosis, but also maintains the stability of STING dimers leading to enhanced inflammatory response, and reduces the nuclear localization of NCOA4, which impairs the transcription factor coregulator function of NCOA4 (30). Additionally, ferroptotic cell debris skews macrophages toward an M1 phenotype, establishing a proinflammatory feed-forward loop (31). M2 macrophages inhibit ferroptosis in themselves and surrounding cells by secreting substances such as cysteine and lactoferrin, and by utilizing their inherent iron metabolism reprogramming and antioxidant programs (32). Inflammatory mediators generated during sepsis also increase intracellular ROS and oxidative metabolites, further exacerbating lung injury. Itaconate, a metabolite produced during inflammatory macrophage activation, inhibits ferroptosis of macrophage via Nrf2 pathways against sepsis-induced ALI/ARDS (33). Similarly, uridine can activate Nrf2 signaling to suppress macrophage ferroptosis (34). Therefore, macrophage ferroptosis plays a crucial role in sepsis-induced ALI, and the inhibition of macrophage ferroptosis may serve as a novel potential therapeutic strategy for ALI/ARDS. Further animal experiments and clinical studies are needed to verify these points. The regulation of the macrophage inflammation by inhibiting ferroptosis to thereby alleviate ARDS may also be a new therapeutic strategy.

### 2.4 Clinical correlation

Emerging clinical evidence supports a link between ferroptosis and sepsis-induced ALI/ARDS severity. Plasma LPO markers such as malondialdehyde and 4-hydroxynonenal (4-HNE), along with ferritin, are elevated in sepsis-induced ALI/ARDS patients and correlate with mortality (35). Reduced GPX4 activity and SLC7A11 expression in bronchoalveolar lavage (BAL) cells predict poor clinical outcomes (36), and GPX4 itself has been proposed as a biomarker of ferroptosis (37). Additionally, iNOS-derived nitric oxide in sepsis S-nitrosylates GPX4, inhibiting its activity. A 2025 study demonstrated that iNOS knockout preserved GPX4 function and reduced ferroptosis in sepsis-induced ALI/ARDS (38). Prostaglandin-Endoperoxide Synthase 2 (PTGS2), as an emerging comprehensive biomarker, has an increase that is a sensitive and quantifiable readout of ferroptosis (3). A 2025 study showed that the PRMT1/EGR1/GLS2 signaling axis drives ferroptosis in sepsis-induced ALI/ARDS. Monitoring the expression levels of PRMT1, EGR1, and GLS2 may provide clues for identifying ferroptosis (39). In addition, GSH, as an important intracellular antioxidant, significantly decreases during ferroptosis (40). Increasingly, ferroptosis (“iron death”) is being recognized as a therapeutic target in S-ARDS. Inhibition of ferroptosis significantly alleviates lung tissue injury by modulating pathways such as GPX4 and FSP1. Thus, ferroptosis inhibitors are emerging as promising therapeutic candidates for sepsis induced ALI/ARDS.

However, the current research has key limitations: most mechanistic studies rely on mouse models, but there are differences in iron metabolism pathways between mice and humans (for example, human TfR1 has a broader tissue expression profile), resulting in the clinical translational value of some targets (such as ACSL4) still needing to be validated. In addition, most studies have not directly focused on the relationship between ferroptosis and ALI/ARDS. Therefore, the direct relationship between them needs to be explored, together with more treatments targeting ferroptosis.

## 3 Therapeutic strategies targeting ferroptosis in sepsis-induced ALI/ARDS

Recent preclinical studies (2020–2025) have validated multiple approaches to inhibit ferroptosis in sepsis-induced ALI/ARDS, many of which exert immunomodulatory effects.

### 3.1 Radical-trapping antioxidants

Ferrostatin-1 (Fer-1) reduces ferroptosis by scavenging lipid-free radicals (8). Its more stable analog liproxstatin-1 (Lip-1) preserves ~55% of alveolar–capillary barrier integrity in LPS-induced sepsis-induced ALI/ARDS (41). A next-generation ferroptosis inhibitor, SRS-11-94, demonstrated 10-fold higher potency and fewer off-target effects than Fer-1 in human alveolar epithelial cultures (42). Nanotechnology-based approaches are also emerging: DQB@C nanosystems alleviate oxidative stress and inflammation in lung cells by upregulating Slc 7a 11/xCT and downregulating Cox 2, thereby regulating ferroptosis (43). Chalcone reduces pulmonary edema by maintaining the integrity of pulmonary vascular endothelial cells and alveolar epithelial cells due to its antioxidant properties and ability to scavenge oxygen-free radicals (44). Irisin treatment also reduces sepsis-induced lung damage and the levels of oxidative stress-related indicators such as ROS and Fe²^+^ (45). Multi-mechanism synergistic interventions indicate that sepsis-induced ALI/ARDS is a complex pathological process involving multiple mechanisms and may become a more effective strategy. In the future, drugs may be combined with different mechanisms to more comprehensively inhibit lung injury through synergy.

### 3.2 Iron chelators

Deferoxamine (DFO) binds unstable iron and reduces iron levels in BAL fluid by 45% when delivered via nebulization in septic rats, effectively alleviating pulmonary edema without causing systemic iron deficiency (46). In multimicrobial sepsis, oral deralarose lowered the levels of ferroptosis markers, such as ACSL4 and 4-HNE, and improved the survival of model animals (47, 48). Targeted iron chelators further improve the intervention effect, such as liposome-encapsulated DFO can be specifically accumulated in lung tissues and its efficacy is 3-fold higher than that of free DFO (49). Moreover, AUF1 inhibits ferroptosis by upregulating NRF2 and downregulating ATF3, thereby reducing sepsis-induced ALI/ARDS (50). Therefore, local targeted iron metabolism is better for avoiding systemic side effects. Optimizing the dosage form of iron chelators can significantly improve the efficacy, indicating that targeted delivery can enhance the specificity and efficiency of intervention.

### 3.3 GPX4 activation and GSH restoration

Selenium (Se), a cofactor of GPX4, restores its activity; supplementation at 0.5 mg/kg increased GPX4 activity by 70% and reduced LPO in sepsis-induced ALI/ARDS mice (51, 52). N-acetylcysteine, a GSH precursor, reduced BAL fluid LPO by 50% and improved respiratory compliance in septic pigs at medium-to-high doses (53). Zyloxadin also boosted GSH levels in human lung endothelial cells exposed to septic plasma, reducing ferroptosis (54, 55).

### 3.4 Combination therapies

The combination of ferroptosis inhibitors with classic anti-inflammatory/antioxidant agents, such as ferroptosis inhibitors (Fer-1/Lip-1) combined with glucocorticoids, can simultaneously block the feedback loop of ‘inflammation promoting ferroptosis’ and ‘ferroptosis exacerbating inflammation,’ achieving synergistic protection (56). In addition, the combination of an iron chelator (deferoxamine, DFO) with glutathione precursors can simultaneously address the two fundamental problems of ‘iron overload’ and ‘antioxidant system failure’ (57). Combining ferroptosis inhibitors with pyroptosis inhibitors can comprehensively alleviate tissue damage and inflammatory responses driven by both ferroptosis and pyroptosis (58).

### 3.5 Novel drugs

Dipyridamole inhibits adenosine uptake, activates bypass signaling, and enhances cystine uptake and glutathione synthesis, thereby effectively suppressing ferroptosis (59). Zileuton reduced LPO by 40% in sepsis-induced ALI/ARDS, with additive effects when combined with Fer-1 (60). Yes-associated protein 1 (YAP1), a Hippo pathway regulator, modulates ferroptosis-related genes and mitigates sepsis-induced ALI/ARDS (10). Srg3 knockdown promotes M2 macrophage polarization, significantly improving sepsis-induced ALI/ARDS outcomes in rats (61). Hydroxychloroquine reduces lung iron accumulation by disrupting the acidic environment of lysosomes, inhibiting the degradation of ferritin and the recycling of iron, and sequestering iron in an inert storage form (62, 63). YAP1, Srg3, and others are involved in ferroptosis regulation, suggesting that ferroptosis-targeted interventions extend beyond traditional iron metabolism and antioxidant frameworks, encompassing signaling pathway cross-talk and immune cell phenotype regulation.

## 4 Conclusions and prospects

Ferroptosis has emerged as a pivotal driver of sepsis-induced ALI/ARDS, with recent studies (2020–2025) clarifying its molecular interplay with immune dysfunction, oxidative stress, and barrier disruption. While ferroptosis-targeted therapies have shown strong preclinical potential against sepsis-induced ALI/ARDS, their clinical translation faces key challenges. The first is the issue of biomarker recognition, which currently lacks validated sepsis-induced ALI/ARDS-specific ferroptosis biomarkers, and GPX4 activity in plasma acylcarnitine and BAL fluid cells is a promising candidate (64, 65). Second, the drug specificity is insufficient, and current ferroptosis inhibitors (RTAs, iron chelators) may affect non-ferroptotic pathways (66, 67). Furthermore, the timing of intervention is critical, as the peak window for ferroptosis is 6–24 h after sepsis onset, and delayed treatment reduces efficacy (68). At the same time, precise drug dosage control is required to avoid side effects, and safer formulations are needed (69, 70). Nonetheless, combination therapies, including those integrating immunotherapy, hold significant promise (71, 72). Future research should prioritize developing reliable biomarkers, optimizing drug delivery systems, and validating combination therapies. Targeting ferroptosis represents a transformative strategy to improve outcomes and reduce mortality in sepsis-induced ALI/ARDS, addressing a long-standing unmet clinical need.
